# Chemical Profiling of an Antimigraine Herbal Preparation, Tianshu Capsule, Based on the Combination of HPLC, LC-DAD-MS^**n**^, and LC-DAD-ESI-IT-TOF/MS Analyses

**DOI:** 10.1155/2014/580745

**Published:** 2014-07-20

**Authors:** Juanjuan Liang, Huimin Gao, Liangmian Chen, Wei Xiao, Zhenzhong Wang, Yongyan Wang, Zhimin Wang

**Affiliations:** ^1^Institute of Chinese Materia Medica, China Academy of Chinese Medical Sciences, Beijing 100700, China; ^2^National Engineering Laboratory for Quality Control Technology of Chinese Herbal Medicine, Beijing 100700, China; ^3^Jiangsu Kanion Pharmaceut Co. Ltd., Lianyungang 222001, China

## Abstract

Chemical profiling is always the first task in the standardization and modernization of Traditional Chinese Medicine. HPLC and LC-MS were employed to find out the common chromatographic peaks in various batches of Tianshu Capsule (TSC) and the contribution of the characteristic peaks from individual herbs to the whole chromatographic profile of TSC sample. A total of 38 constituents were identified in TSC sample based on the comparison of retention time and UV spectra with authentic compounds as well as by summarized MS fragmentation rules and matching of empirical molecular formula with those of published components. This is the first systematic report on the chemical profiling of the commercial TSC product, which provides the sufficiently chemical evidence for the global quality evaluation of TSC products.

## 1. Introduction

Traditional Chinese Medicine (TCM) is getting more attention all over the world due to its exact clinical practice, especially prescription application, which comprehensively highlights the quintessence of the theory of traditional Chinese medical science.* Da Chuan Xiong Fang* (DCXF), a well-known and extensively used TCM decoction for the treatment of migraine, first appeared in* Xuan Ming Lun Fang,* a famous formula book written by Wansu Liu who lived in Jin Dynasty (1115–1234). It is composed of two herbs, namely, Chuanxiong (*Chuanxiong rhizoma*) and Tianma (*Gastrodiae rhizoma*), with a crude weight ratio of 4 : 1. Eight dosage forms of DCXF such as capsule, tablet, dripping-pill, honeyed pill, oral liquid, and granule, have been authorized to Chinese market. Tianshu Capsule (TSC), as a representative of DCXF preparations, is widely used in clinics for treating the blood stasis type of headache and migraine [1, 2].

Phytochemical and pharmacological investigations showed that phenols, organic acids, phthalides, and nitrogen-containing compounds were the major active ingredients of DCXF [3]. Several qualitative analyses have been reported concerning main types of constituents in DCXF [4–7]. One study described the identification of 17 different constituents in the 50% EtOH extract of DCXF by LC-Q-TOF/MS, containing gastrodin, parishin C, ferulic acid, guanosine, adenosine, palmitic acid, and 11 phthalide compounds [4]. Another similar study identified 3 compounds of Chuanxiong (ferulic acid, senkyunolide I, and senkyunolide H) and 8 constituents of Tianma (gastrodin, s-(4-hydroxybenzyl)-glutathione, parishin, parishin B, parishin C, p-hydroxybenzaldehyde, etc.) by using the HPLC-DAD-MS^n^ coupling technique, respectively [5]. Continuous reports from the second research group confirmed that 10 different compounds, including 6 original substances of Chuanxiong and 4 original ones of Tianma, were detected in the rat plasma after the gavage of DCXF active components [6, 7]. However, all these investigations mentioned above were carried out based on the samples of the 50% EtOH extract of the mixture of both herbs (4 : 1) or active components of single crude herb. No systematic reports could be available involving the chemical profiling of the commercial finished products derived from DCXF. TSC was produced from both crude herbs by employing the various pharmaceutical engineering technologies and the complex manufacturing processes such as extraction, concentration, and preparation. The accumulating studies showed that decocting could induce chemical changes of medicinal herbs or combinatorial formula [8]. It is well-known that ferulic acid and some of the phthalides such as* Z*-ligustilide and dimeric phthalide are unstable at high temperature [9, 10]. Therefore, during the preparation of TSC, these thermolabile components may undertake chemical transformation, consequently leading to the difference of chemical compositions of finished products with DCXF decoction. Understanding the chemical profiles of TSC samples would be helpful in selecting suitable chemical markers for the quality control and pharmacokinetic study. In this work, a combination of HPLC, LC-DAD-MS^n^, and LC-DAD-ESI-IT-TOF/MS analyses was employed to find out and identify the common components in various batches of commercial TSC samples. The contribution of the characteristic peaks from individual herb to the whole chemical profiling of TSC was also discussed. A total of 38 constituents were identified or tentatively characterized, among which the water-soluble compounds with higher polarity from* Gastrodiae rhizoma* are detected in TSC samples for the first time.

## 2. Materials and Methods

### 2.1. Materials and Reagents

**Figure 1:**
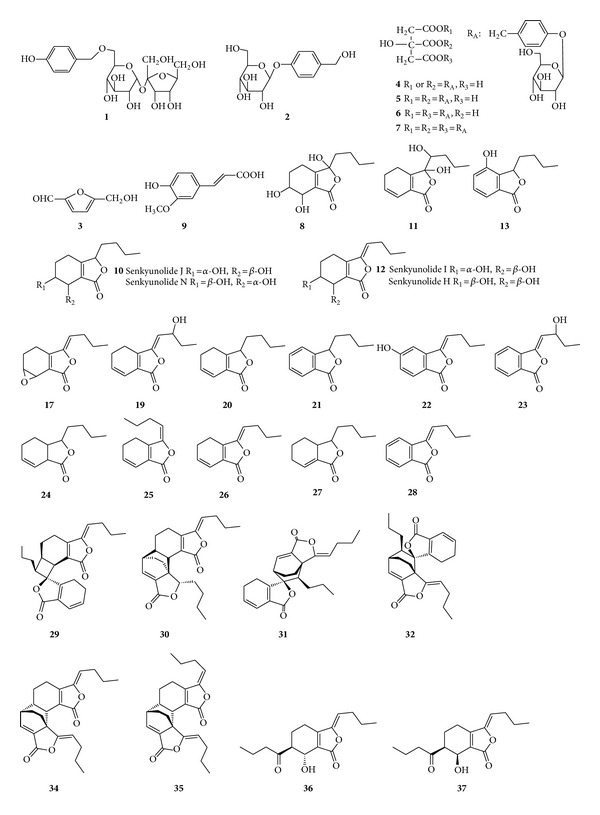
The structures of the identified compounds in TSC sample.

Five batches of Tianshu Capsules and related crude herbal materials (*Chuanxiong rhizoma and Gastrodiae rhizoma*) were provided by* Kanion Pharmaceutical *(Lianyungang, China). The reference substances of gastrodin (Lot. 110807-200205), 5-hydroxymethyl-2-furfural (5-HMF, Lot. 111626-201007), ferulic acid (Lot. 0773-9607), and* Z*-ligustilide (Lot. 111737-201102) were purchased from the National Institutes for Food and Drug Control (Beijing, China). Parishin B (174972-79-3), parishin C (174972-80-6), and parishin (62499-28-9) were from the collection of Dr. Li Wang, Dalian Institute of Chemical Physics, Chinese Academy of Sciences (Dalian, China). The purity for each of these compounds was over 98% by HPLC assay and their structures were shown in Figure 1. HPLC grade methanol (Fisher, Fair Lawn, NJ, USA) and ultrapure water were used for HPLC analyses. All other chemical reagents were of analytical grade from Beijing Chemical Corporation (Beijing, China).

### 2.2. Sample Preparation

#### 2.2.1. TSC Sample

1.0 g of pulverized contents of TSC samples was extracted with aqueous methanol (MeOH-H_2_O, 1 : 1, 25 mL) by ultrasonication (250 w, 40 kHz) for 30 min at room temperature, and the extract was then centrifuged for 10 min at 14800 rpm. A volume of 10 *μ*L of the supernatant was used for HPLC and LC-DAD-MS^n^ analysis.

#### 2.2.2. Extraction of Chuanxiong Rhizoma, Gastrodiae Rhizoma, and DCXF

The ethanolic and aqueous extracts of individual herb (*Chuanxiong rhizoma* and* Gastrodiae rhizoma*) and the mixture of both herbs (w/w, 4 : 1, DCXF) were prepared according to the manufacturing processes of TSC (Figure S1 in the Supplementary Material available online at http://dx.doi.org/10.1155/2014/580745) described in the current Chinese pharmacopoeia. The ethanolic and aqueous extracts were diluted with aqueous methanol (MeOH-H_2_O, 1 : 1) to the concentration of 0.05 g·mL^−1^ and then centrifuged for 10 min at 14800 rpm. Each of the supernatants was used for HPLC and LC-MS analyses.

#### 2.2.3. Reference Solution

Stock solutions with a concentration of about 0.010 mg·mL^−1^ were prepared by dissolving an accurately weighed amount of each reference substance in aqueous methanol (MeOH-H_2_O, 1 : 1). The mixture of 7 reference solutions was prepared from the stock solutions.

### 2.3. Qualitative HPLC Analyses of 5 Batches of TSC Samples

The analyses were performed on a Shimadzu HPLC system (Shimadzu, Japan) equipped with a LC-20AT binary pump, a DGU-20A5 degasser, a SIL-20AC autosampler, a CTO-20AC column oven, and a SPD-M20A photodiode array detector. The samples were separated on a Phenomenex Luna C_18_ column (5 *μ*m, 4.6 × 250 mm). The mobile phase consisted of methanol (A) and water containing 0.1% formic acid (B) using a gradient program as follows: 0 min, 15% A; 5 min, 15% A; 55 min, 95% A; 60 min, 95% A. The flow rate was 1.0 mL·min^−1^ and the column temperature was set at 30°C. The PDA detector recorded UV spectra in the range from 190 nm to 400 nm and HPLC chromatogram was monitored at 276 nm.

### 2.4. Comparison of Typical TSC Sample and Its Related Crude Herbal Materials as Well as DCXF by HPLC and LC-MS

TSC sample and its related crude herbal material,* Chuanxiong rhizoma *and* Gastrodiae rhizoma *as well as DXCF, were analyzed under the same chromatographic conditions by HPLC and LC-MS to find the contribution of individual herb to the whole chemical profile of TSC sample. The HPLC system was the same as those in Section 2.3. LC-MS analyses were performed using an Agilent 6130 Quadrupole LC-MS (Agilent, Waldbronn, Germany) connected to an Agilent 1200 HPLC system (Agilent, Waldbronn, Germany). The parameters for MS analysis in the positive and negative ion mode were as follows: nebulizer, 35 psi; ionization voltage, 3500 V; dry temperature, 350°C; flow rate of carrier gas, 9.0 L·min^−1^. Full-scan mass spectra were acquired in the range of 100–800* m/z*.

### 2.5. LC-DAD-ESI-IT-MS^n^ Analysis of Typical TSC Sample

To comprehensively identify the chemical constituents in TSC sample by the fragmentation rules, a LC-DAD-ESI-IT-MS^n^ experiment was performed using an Agilent 6320 ion-trap spectrometer (Agilent, Waldbronn, Germany) connected to an Agilent 1200 HPLC system (Agilent, Waldbronn, Germany). The HPLC conditions were the same as those described in Section 2.3. The LC effluent was introduced into an electrospray ionization source after a postcolumn split ratio of 2 : 1. The parameters for MS analysis in the positive ion mode were as follows: nebulizer, 45 psi; ionization voltage, 4000 V; dry temperature, 350°C; flow rate of carrier gas, 12.0 L·min^−1^. Full-scan mass spectra were acquired in the range of 100–800* m/z*. The optimized parameters for MS/MS analysis were as follows: collision energy, 1.5 V; nitrogen was used as the collision gas. MS^n^ spectra of pure substances were obtained using the same parameters as mentioned above.

### 2.6. LC-DAD-ESI-IT-TOF/MS of Analysis of Typical TSC Sample

To confirm the elemental composition of precursor ions and their fragments with high-accurate mass, a LC-ESI-IT-TOF/MS experiment was performed on a Shimadzu LC-MS-IT-TOF instrument equipped with a Shimadzu UFLCXR HPLC system (Shimadzu, Kyoto, Japan). The HPLC system consisted of a CBM-20A controller, two LC-20AD binary pumps, an SPD-M20A diode array detector, an SIL-20AC autosampler, a CTO-20A column oven, and a DGU-20A3 degasser. The HPLC conditions were the same as those for HPLC-DAD-ESI-MS^n^ analysis. The LC effluent was directed into the ESI source as a rough split ratio of 3 : 1. The optimized MS conditions were as follows: positive and negative ion mode; electrospray voltage, +4.5 kV/−3.5 kV; detector voltage, 1.65 kV; curved desolvation line (CDL) temperature, 200°C; heat block temperature, 200°C; nebulizing gas (N_2_), 1.5 L·min^−1^; drying gas (N_2_), 10 L·min^−1^; scan range,* m/z* 100–1100 for MS^1^, 100–800 for MS^2^, and 100–500 for MS^3^. The ultrahigh purity argon was used as the collision gas for collision-induced dissociation (CID) experiments, and the collision energy was set at 50% for MS^2^ and MS^3^; ion accumulated time was 30 ms. The MS^n^ data were collected in an automatic mode and the software could automatically select precursor ions for MS^n^ analysis according to criteria settings. Accurate mass determination was corrected using the external standard method. The data acquisition and analysis were performed by LC-MS Solution Version 3.6 software (Shimadzu, Kyoto, Japan).

## 3. Results and Discussion

### 3.1. Qualitative Analyses of TSC and Its Related Crude Herbal Materials by HPLC and LC-MS

Under the HPLC conditions as described in the current Chinese Pharmacopoeia [1], 5 batches of TSC samples, together with the reference compounds, were examined and their HPLC chromatograms were shown in Figure S2. High similarity in the number, type, and amount of chemical constituents was observed in the HPLC profiles of different batches of TSC samples. General chromatographic profile was obtained by Similarity Evaluation System for Chromatographic Fingerprint of Traditional Chinese Medicine software and characteristic peaks were found in the HPLC profile of each individual sample.

**Figure 2:**
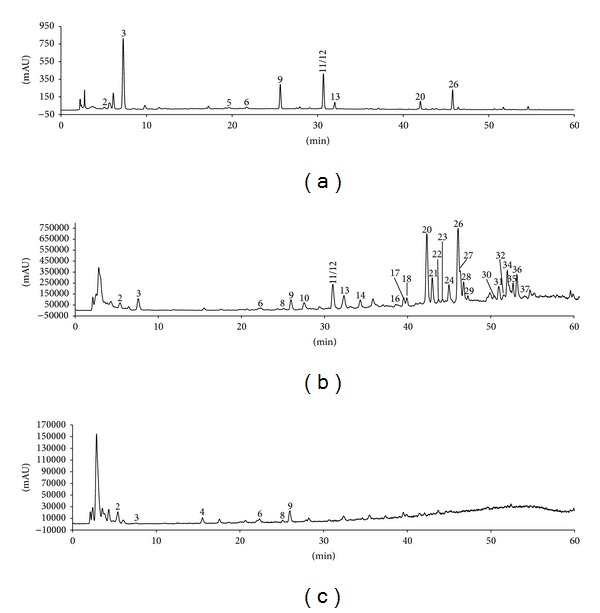
HPLC and TIC of typical TSC sample obtained using an Agilent 6130 Quadrupole LC-MS connected to an Agilent 1200 HPLC system. (a) HPLC (276 nm), (b) (+) TIC, and (c) (−) TIC.

In order to identify the origin of these characteristic peaks from individual herbs, a comparative study was carried out by using various extracts of herbs and TSC samples. Accordingly, the possible individual contribution from the corresponding herbs to the general chromatographic profile was found. Compared with the HPLC profiles of the ethanolic and aqueous extracts of* Chuanxiong rhizoma and Gastrodiae rhizome* (Figure S3), 29 of 38 peaks occurring in HPLC profile of TSC sample were contributed by* Chuanxiong rhizoma* and other 9 peaks came from G*astrodiae rhizome* (Figures S4 ~ S10). In addition to the comparison of retention time and on-line UV spectra with those of reference compounds gastrodin, 5-HMF, parishin B, parishin C, parishin, ferulic acid, and* Z*-ligustilide, the precursor ions obtained by the positive and negative LC-MS (Figure 2) such as [M+H]^+^, [M+NH_4_]^+^, [M+Na]^+^, and [M-H]^−^ further confirmed the contribution of the characteristic peaks from individual herbs to the chromatographic profile of TSC sample (Table 1).

### 3.2. Identification of Chemical Constituents in TSC by LC-DAD-EST-IT-MS^n^ and LC-DAD-ESI-IT-TOF/MS

The combination of LC-DAD-ESI-IT-MS^n^ and LC-DAD-ESI-IT-TOF/MS experiments was employed for the identification of chemical constituents in TSC sample, and, as a result, a total of 38 compounds was identified or tentatively characterized. The structures of the identified compounds are shown in Figure 1 and their chromatographic and mass spectrometric data are shown in Tables 2 and 3. The total ion chromatograms (TICs) of TSC sample are shown in Figures S11 and S12, respectively. Among the identified constituents, 7 compounds (**2**,** 3**,** 5**,** 6**,** 7**,** 9,** and** 26**) were unambiguously identified as gastrodin, 5-HMF, parishin B, parishin C, parishin, ferulic acid, and* Z*-ligustilide based on the direct comparison of their retention times, UV spectra, and mass spectra with those of the authentic compounds. Furthermore, 31 compounds were tentatively characterized according to their UV spectra, empirical molecular formula, and mass fragmentation pathways as well as their eluted sequence from ODS column reported in the literature [11–14] and acquired in the present experiments.

#### 3.2.1. Fragmentation Characteristic of Reference Compounds

In the present HPLC and MS conditions, characteristic MS adduct ions were observed for phenolic glycosides, organic acid, and phthalide derivatives. Phenolic glycosides and organic acids could be well-detected in positive and negative ionization mode and adduct ions such as [M+H]^+^, [M+NH_4_]^+^, [M+Na]^+^, and [2M+Na]^+^ or [M-H]^−^ and [M+HCOO]^−^ were found, whereas phthalides were only detected in positive mode and mainly showed the abundant [M+H]^+^, [M+Na]^+^, and [2M+Na]^+^ ions. The fragmentation characteristic of reference compounds was similar as those described in the literature [11, 14, 15]. For example, gastrodin, the glucoside of* p*-hydroxybenzyl alcohol, mainly showed characteristic product ions at* m/z *123 and 161 corresponding to the elimination of a molecule of glucose and a molecule of* p*-hydroxybenzyl alcohol from* m/z* 285 [M-H]^−^, respectively. Parishin B and C, the gastrodin derivatives with citric acid, mainly indicated the fragmentation of the ester glucoside bond and the neutral loss of gastrodin residue (268 Da) corresponding to the ion at* m/z* 459. The ions at* m/z *441, 423, 397, and 379 were also observed, which were related to elimination of H_2_O and CO_2_ from the tertiary alcoholic hydroxyl group and the free carboxylic groups produced by breaking of the ester glucoside bond. Phthalides mainly displayed two pathways: side-chain cleavage with loss of alkenes and ring-opening followed by elimination of H_2_O and CO. These series of characteristic ion rules would be beneficial to elucidate the chemical constituents in TSC sample.

#### 3.2.2. Identification of Phenolics Derivatives in TSC Sample

Compounds** 1** and** 2** gave the same on-line UV spectrum which is in accordance with that of gastrodin. The structure of** 2** was identified as gastrodin based on the comparison of retention time, UV spectrum, and characteristic fragment ions with those of authentic compound as well as accurate molecular weight. The molecular weight of** 1** was deduced as 448 from the sodium adduct ion at* m/z* 471 [M+Na]^+^ detected in positive mode and deprotonated molecular ion at* m/z* 447 [M-H]^−^ in negative mode, respectively. A prominent neutral loss of 162 Da, corresponding to the loss of hexose, and disaccharide residue ions at* m/z* 323 were observed in MS^2^ of the [M-H]^−^ ion at* m/z* 447. The loss of 124 Da was assigned to* p*-hydroxybenzyl alcohol just like the characteristic loss of gastrodin. Therefore, compound** 1** was identified as elatoside, namely, 6′-(*p*-hydroxybenzyl methyl)-gastrabiose, which was previously isolated from the rhizome of* G*.* elata *[16].

Compounds** 4**,** 5**,** 6,** and** 7** gave the adduct ions [M+Na]^+^ and [M+NH_4_]^+^ in positive LC-MS and [M-H]^−^ in negative LC-MS experiments as well as UV spectra, which were similar to those of gastrodin derivatives. Compounds** 5** and** 6** had the same [M-H]^−^ ions at* m/z* 727, which produced the prominent fragment ion at* m/z* 459 in the MS^2^, due to loss of gastrodin residue (268 Da) as well as other ions at* m/z *441, 423, 397, and 379. Combining with retention time and addition of reference substances,** 5** and** 6** were identified as parishin B and C. Similarly, compound** 4 **was assigned to parishin E or its positional isomer parishin G with identical molecular mass of 460 [17]. Compound** 7** had the molecular mass of 996 and exhibited consecutive loss of gastrodin residue (268 Da), and, therefore, it was identified as parishin, a conjugate of one citric acid and three gastrodins.

#### 3.2.3. Identification of Phthalide Derivatives in TSC Sample

The molecular formula of compound** 8**, detected from individual herb* Chuanxiong rhizoma*, was calculated as C_12_H_18_O_5_ by [M+H]^+^ ion at* m/z* 243.1220 and [M-H]^−^ ion at* m/z* 241.1090 in its LC-IT-TOF/MS experiment. It had the same molecular weight of 242 as those of known compounds, senkyunolide L and 3-butyl-3,6,7-trihydroxy-4,5,6,7-tetrahydrophthalide, present in* Chuanxiong rhizoma*; however, compound** 8** could not be senkyunolide L (C_12_H_15_ClO_3_) because both had the different element composition [18]. The fragmentation ion of** 8** at* m/z* 197.1142, which was derived from a retro-Diels-Alder cleavage of the [M-H-H_2_O]^−^ ion at* m/z* 223.0885, suggested that the structure of** 8** was proposed as 3-butyl-3,6,7-trihydroxy-4,5,6,7-tetrahydrophthalide.

Compound** 10** displayed [M+H]^+^ ion at* m/z* 227.1259, suggesting the molecular formula of C_12_H_18_O_4_. The fragment ions at* m/z* 209 and 191 in the MS^2^, indicating consecutive loss of H_2_O, supported that** 10** was a dihydroxylated derivative of ligustilide. UV and MS data of** 10** were in accordance with those of senkyunolide J and senkyunolide N, but the stereochemistry information of two hydroxy groups could not be provided. Thus,** 10** was tentatively assigned as one of senkyunolide J and senkyunolide N. Compounds** 11** and** 12** gave the same [M-H]^−^ ions at* m/z* 224 and their molecular formula was established as C_12_H_18_O_4_ according to the [M+H]^+^ ion at* m/z* 225.1105. However, both constituents had different fragmentation rules. The MS^2^ of** 11** displayed the fragment ions at* m/z* 207, 189 and the characteristic ion at* m/z* 165, which were similar to those of 4,5-dihydro-3,1′-dihydroxy-3-butylphthalide [15], whereas** 12** showed elimination of two H_2_O followed by side-chain cleavage. Therefore,** 11** was deduced as 4,5-dihydro-3,1′-dihydroxy-3-butylphthalide and** 12** could be one of senkyunolide I and senkyunolide H [14, 15].

Compounds** 13**,** 17,** and** 19** have the same molecular formula of C_12_H_14_O_3_ and were tentatively characterized as 4-hydroxy-3-butylphthalide,* Z*-6,7-epoxyligustilide, and senkyunolide F. Compared with monohydroxy phthalide derivatives** 13** and** 19**, the instable structure of** 17** resulted in the ion at* m/z* 189 as base peak observed in MS^1^, suggesting loss of H_2_O from the [M-H]^−^ ion at* m/z* 207. Senkyunolide A** 20** and* Z*-ligustilide** 26** as main constituents were identified and butylphthalide** 21**, senkyunolide C** 22**, senkyunolide E** 23**,* E*-ligustilide** 24,** and 3-butylidenephthalide** 28** were characterized as minor constituents. For the compounds** 20**,** 21**,** 24,** and** 26**, the neutral loss of CO (28 Da) and side-chain cleavage (56 Da) from [M+H-H_2_O]^+^ were the common fragmentation rules.

Compound** 16** (C_12_H_14_O_3_) was tentatively proposed to 4,7-dihydroxy-3-butylidenephthalide or senkyunolide D. Compound** 18** was assigned as senkyunolide K or senkyunolide G. These compounds and their isomers could not be differentiated by available MS data.

Five dimeric ligustilides** 29**,** 31**,** 32**,** 34,** and** 35**, which produced the protonated ion [M+H]^+^ at* m/z* 381, were detected in extracted ion chromatograms. They showed the base peak at* m/z* 403 [M+Na]^+^ in MS^1^ and the fragment ion at* m/z* 191 [M+H-190]^+^ as base peak in MS^2^ by the loss of a RDA fragment (145 Da) followed by the loss of H_2_O and HCOOH (46 Da). Comprehensively considering the information described in the literature [14], they were tentatively identified as riligustilide, 3′,6,8′,3a-biligustilide, tokinolide B, levistolide A, and senkyunolide O, respectively. Three compounds, indicating the protonated ion [M+H]^+^ at* m/z *383, were detected in extracted ion chromatograms; however, only senkyunolide P was previously reported in* Chuanxiong Rhizoma*. Thus, compound** 30** was tentatively characterized as senkyunolide P and the other compounds (**33** and** 38**) were still indefinite based on the available information.

In the modernization of TCM, chemical profiling is always the first task. It is of importance for development of the suitable quality standard and control strategy, study of pharmacokinetics, and interpretation of therapeutic character of TCM [19, 20]. As a marketed product in China, the quality control system of TSC is still to be improved and its pharmacokinetics and action mechanism are not completely clear. Usually, gastrodin, ferulic acid, and 6,7-dihydroxyligustilide are selected as marker compounds for the quality control or the pharmacokinetic study of Tianshu Capsule [1, 21, 22]. The present investigation provides more chemical information for the selection of the marker compounds and the improvement of quality control. It also tells the pharmacokinetic scientists and ethnopharmacologists which compounds in TSC samples are worth further evaluation. In addition to gastrodin and ferulic acid, more efforts should be made to the group of phthalide derivatives such as major constituents, senkyunolide I/H** 12**, 4-hydroxy-3-butylphthalide** 13**, senkyunolide A** 20,** and* Z*-ligustilide** 26** as well as their dimers. Additionally, it is worth noting that 5-HMF** 3**, a potent toxic compound, was found as main peak in the chemical profile of TSC samples. This compound originated from the crude material* Gastrodiae rhizoma *and its content in TSC samples was up to 2.67 mg·g^−1^ [23]. If it is necessary to develop its limit standard in TSC product or not, more study are expected. Also, ligustrazine is not detected in TSC sample, even in the extracted ion chromatogram, which could result from its very low amount in crude herbal material (about 0.01~0.02%), although, actually, it is often considered as one of active components in* Chuanxiong rhizoma*.

## 4. Conclusions

In this study, HPLC analysis was employed to find out the common chromatographic peak in various batches of TSC samples. The contribution of the characteristic peaks from individual herbs to the whole chromatographic profile was discussed based on comparative HPLC and LC-MS analyses. A total of 38 constituents were identified based on the comparison of retention time and UV spectra with authentic compounds as well as by summarized MS fragmentation rules and matching empirical molecular formula with those of published components. The present investigation provided the good basis for monitoring the manufacturing processes and improving quality control of TSC products.

## Supplementary Material

Figure S1 The pharmaceutical manufacture process of TSC described in current Chinese Pharmacopoeia
Figure S2. HPLC of 5 batches of TSC samples at 276 nm
Figure S3. HPLC of reference solution (RS) and TSC sample. A. RS at 276 nm, B. RS at 221 nm, C. TSC sample at 276 nm, D. TSC sample at 221 nm.
Figure S4. HPLC of ethanolic (e) or aqueous (a) extracts of *Da Chuanxiong Fang* (D.), *chuanxiong rhizoma* (C.) and *gastrodiae rhizoma* (G.) at 276 nm.
Figure S5. HPLC and TIC of ethanolic extract of *Da Chuanxiong Fang*. A. HPLC (276 nm), B. (+) TIC, C. (-) TIC.
Figure S6. HPLC and TIC of aqueous extract of *Da Chuanxiong Fang*. A. HPLC (276 nm), B. (+) TIC, C. (-) TIC.

## Figures and Tables

**Table 1:** Retention time (*t*_*R*_), UV, and LC-MS data of the identified compounds in Tianshu Capsule and related crude materials.

Number	*t*_*R*_/min	UV *λ*_max⁡_/nm	MW	[M − H]^−^	[M + H]^+^	[M + NH_4_]^+^	[M + Na]^+^	[2M + Na]^+^	[M + HCOO]^−^	Tianma	Chuanxiong	Identified compounds
**2**	5.403	221, 276	286			304			331	+	−	Gastrodin
**3**	7.736	230, 283	126		127	144	149			+	−	5-HMF
**4**	15.579	225	460	459		478	483			+	−	Parishin E/G
**5**	20.691	221, 272	728	727		746	751			+	−	Parishin B
**6**	22.220	221, 274	728	727		746	751			+	−	Parishin C
**8**	25.080	218	242	241	243		265	507		−	+	3-Butyl-3,6,7-trihydroxy-4,5,6,7 -tetrahydrophthalide
**9**	25.567	220, 236, 323	194	193	195		217			+	+	ferulic acid
**10**	27.529	220, 330	226		227		249	475		−	+	Senkyunolide J/N
**11**	30.667	280	224		225		247	471		−	+	4,5-Dihydro-3,1′-dihydroxy-3 -butylphthalide
**13**	32.436	275	206	205	207					−	+	4-hydroxyl-3-butylphthalide
**14**	34.299	325	396		397					+	+	unidentified
**16**	38.635	220, 270	222	221	223		245			−	+	Senkyunolide D or 4,7-dihydroxy-3-butylidenephthalide
**17**	39.502	230, 310	206	205	207		229		435	−	+	*Z*-6,7-Epoxyligustilide
**18**	39.898	280	208	207	209		231		439	−	+	Senkyunolide K/G
**19**	41.465	220	206	205	207		229		435	−	+	Senkyunolide F
**20**	42.332	220, 240, 325	192		193		215	407		+	+	Senkyunolide A
**21**	42.956	226, 280	190		191	208	213	403		−	+	Butylphthalide
**22**	43.717	218, 270	204	203	205		227	431	249	−	+	Senkyunolide C
**23**	44.147	210, 285, 330	204	203	205		227	431	249	−	+	Senkyunolide E
**24**	44.949	220, 270, 330	194		195		217	411		−	+	Cnidilide
**26**	45.831	205, 280, 328	190		191		213	403		−	+	Ligustilide
**27**	46.394	220	194		195		217	411		−	+	Neocnidilide
**28**	46.653	210, 275, 330	188		189	206	211	399		−	+	3-Butylidenephthalide
**29**	47.231	225, 280, 310	380		381	398	403			−	+	Riligustilide
**30**	50.042	276	382		383	399	405			−	+	Senkyunolide P
**31**	50.476	220, 280	380		381	398	403			−	+	3′,6,8′,3a-Biligustilide
**32**	50.970	220, 280	380		381	398	403			−	+	Tokinolide B
**33**	51.065	230, 279	382		383	400	405			−	+	Unidentified
**34**	51.962	220, 275	380		381		403			−	+	Levistolide A
**35**	52.267	220, 285	380		381					−	+	3′*Z*-3,3′a,8,6′a-Biligustilide
**36**	52.693	220, 280	278		279	301	579			−	+	Senkyunolide M
**37**	53.134	220, 280	278		279	301	579			−	+	Senkyunolide Q
**38**	53.852	225, 278	382		383	400	405	383		−	+	Unidentified

**Table 2:** Retention time (*t*_*R*_), UV, and MS^n^ data obtained by LC-DAD-ESI-IT-MS^n^ of the identified compounds in Tianshu Capsule.

Number	*t*_*R*_/min	Identified compounds	UV *λ*_max⁡_/nm	[M + Na]^+^	[M + H]^+^	[2M + Na]^+^	Main product ions	[M − H]^−^	[M + HCOO]^−^	Main product ions
**1**	6.3	Gastrodin glucose (448)	221, 276	471				447	493	323[M – H-C_7_H_8_O_2_]^−^, 285[M – H-glc]^−^, 179
**2**	6.3	Gastrodin (286)	220, 269	309			185[M + Na-C_7_H_8_O_2_]^+^	285	331	161[M – H-C_7_H_8_O_2_]^−^, 123[M – H-glc]^−^
**3**	8.1	5-HMF (126)	230, 283		127		109[M + H-H_2_O]^+^	125		
**5**	20.3	Parishin B (728)	221, 272	751			483[M + Na-268]^+^, 215[483-268]^+^	727		459, 441, 423, 397, 379, 217
**6**	21.8	Parishin C (728)	221, 274	751			483[M + Na-268]^+^, 215[483-268]^+^	727		459, 441, 423, 397, 379, 217
**7**	23.4	Parishin (996)	221, 271					995		727[M – H-268]^−^
**9**	26.0	Ferulic acid (194)	220, 236, 323		195		177[M + H-H_2_O]^+^, 145[177-CH_3_OH]^+^			
**10**	27.4	Senkyunolide J/N (226)	220, 330	249		475	209[M + H-H_2_O]^+^, 191[M + H-2H_2_O]^+^, 163[M + H-2H_2_O-28]^+^			
**11**	30.4	4,5-Dihydro-3,1′-dihydroxy -3-butylphthalide (224)	280	247			207[M + H-H_2_O]^+^, 189[M + H-2H_2_O]^+^, 165, 121			
**12**	31.6	Senkyunolide I/H (224)	276	247			207[M + H-H_2_O]^+^, 189[M + H-2H_2_O]^+^, 161[189-H_2_O]^+^, 133[189-56]^+^			
**15**	37.0	Unidentified (316)	230, 276, 280	339	317		299[M + H-H_2_O]^+^, 281[M + H-2H_2_O]^+^, 271[M + H-H_2_O-28]^+^, 243	315		
**17**	37.8	*Z*-6,7-Epoxyligustilide (206)	230	229		413	189[M + Na-H_2_O]^+^, 171[189-H_2_O]^+^			
**18**	38.1	Senkyunolide K/G (208)	233, 280	231		439	191[M + H-H_2_O]^+^, 173[191 − H_2_O]^+^, 149[191-42]^+^, 135[191-56]^+^			
**20**	40.2	Senkyunolide A (192)	280	215	193	407	175[M + H-H_2_O]^+^, 147, 119, 105			
**21**	40.7	Butylphthalide (190)	232, 275	213	191	403	173[M + H-H_2_O]^+^, 145, 117			
**24**	42.6	Cnidilide (194)	238, 279, 327	217						
**25**	43.3	*E*-Ligustilide (190)	281, 327	213	191	403	173[M + H-H_2_O]^+^, 130			
**26**	43.5	*Z*-Ligustilide (190)	237, 260, 312	213	191	403	173[M + H-H_2_O]^+^, 145[173-28]^+^ 130, 117, 105			
**28**	44.0	3-Butylidenephthalide (188)	230, 277, 326	211	189		171[M + H-H_2_O]^+^, 153[171-H_2_O]^+^			
**29**	47.4	Riligustilide (380)	275	403	381		213[2M + Na-190]^+^, 191, 173			
**30**	47.5	Senkyunolide P (382)	278	405	383		192[M + H-190]^+^			
**31**	47.8	3′,6,8′,3a-Biligustilide (380)	278, 363	403	381		213[2M + Na-190]^+^, 191[M + H-190]^+^, 145, 117			
**32**	48.0	Tokinolide B (380)	278	403	381		213[2M + Na-190]^+^, 191[M + H-190]^+^			
**33**	48.2	Unidentified (382)	230, 279	405	383		193[M + H-190]^+^			
**34**	48.5	Levistolide A (380)	276	403	381		213[2M + Na-190]^+^, 191[M + H-190]^+^, 145, 117			
**35**	48.8	Senkyunolide O (380)	278	403	381		173[191-H_2_O]^+^, 191[M + H-190]^+^, 145, 117			
**36**	49.0	Senkyunolide M (278)	279	301	279		245[M + Na-56]^+^, 189, 171			
**37**	49.4	Senkyunolide Q (278)	278	301	279		245[M + Na-56]^+^, 189, 171			
**38**	49.9	Unidentified (382)	225, 278	405	383					

**Table 3:** Retention time (*t*_*R*_) and MS data obtained by LC-DAD-ESI-IT-TOF/MS of the identified compounds in the sample of Tianshu Capsule.

Number	*t*_*R*_/min	Identified compounds	Formula	Mea. mass/*m*/*z*	Calc. mass/*m*/*z*	Error/ppm	Other precursor ions	Main product ions
**3**	7.6	5-HMF (126)	C_6_H_6_O_3_	127.0384[M + H]^+^	127.0390[M + H]^+^	−4.72	149.0240[M + Na]^+^	
**5**	19.8	Parishin B (728)	C_32_H_40_O_19_	727.2123[M − H]^−^	727.2091[M − H]^−^	4.40		
**6**	21.5	Parishin C (728)	C_32_H_40_O_19_	727.2132[M − H]^−^	727.2091[M − H]^−^	5.64		
**8**	24.5	3-Butyl-3,6,7-trihydroxy-4,5,6,7-tetrahydrophthalide (242)	C_12_H_18_O_5_	241.1090[M − H]^−^	241.1081[M − H]^−^	3.72		223.0885[M – H-H_2_O]^−^, 197.1142, 179.1107, 141.0930, 123.0854
				243.1220[M + H]^+^	243.1227[M + H]^+^	2.88	265.1060[M + Na]^+^	
**9**	25.3	Ferulic acid (194)	C_10_H_10_O_4_	195.0649[M + H]^+^	195.0652[M + H]^+^	−1.54	217.0458[M + Na]^+^	
**10**	27.0	Senkyunolide J/N (226)	C_12_H_18_O_4_	227.1259[M + H]^+^	227.1278[M + H]^+^	−3.37	249.1082[M + Na]^+^	209.1177[M + H-H_2_O]^+^, 191.1026[M + H-2H_2_O]^+^
**11**	30.3	4,5-Dihydro-3,1′-dihydroxy-3-butylphthalide (224)	C_12_H_16_O_4_	225.1105[M + H]^+^	225.1121[M + H]^+^	−7.11		
**12**	31.6	Senkyunolide I/H (224)	C_12_H_16_O_4_	225.1105[M + H]^+^	225.1121[M + H]^+^	−7.11		
**15**	37.8	Unidentified (316)	C_18_H_20_O_5_	317.1380[M + H]^+^	317.1384[M + H]^+^	−1.26	339.1210[M + Na]^+^	299.1378[M + H-H_2_O]^+^, 271.1343[M + H-H_2_O-28]^+^
**16**	37.8	4,7-Dihydroxy-3-butylidenephthalide or senkyunolide D (222)	C_12_H_14_O_4_	223.0986[M + H]^+^	223.0965[M + H]^+^	9.41	245.0774[M + Na]^+^	
**17**	38.7	*Z*-6,7-Epoxyligustilide (206)	C_12_H_14_O_3_	207.1008[M + H]^+^	207.1016[M + H]^+^	3.66	229.0824[M + Na]^+^	189.0856[M + H-H_2_O]^+^, 133.0318
**18**	39.1	Senkyunolide K/G (208)	C_12_H_16_O_3_	209.1167[M + H]^+^	209.1172[M + H]^+^	−2.39	231.0974[M + Na]^+^	191.1091[M + H-H_2_O]^+^, 119.0819
**19**	40.6	Senkyunolide F (206)	C_12_H_14_O_3_	207.1009[M + H]^+^	207.1016[M + H]^+^	3.38	229.0815[M + Na]^+^	189.0911[M + H-H_2_O]^+^, 161.0959
				205.0874[M − H]^−^	205.0870[M − H]^−^	1.95	411.1821[2M − H]^−^	161.0976[M – H-44]^−^, 106.0421
**20**	41.4	Senkyunolide A (192)	C_12_H_16_O_2_	193.1219[M + H]^+^	193.1223[M + H]^+^	−2.07	215.1027[M + Na]^+^, 407.2196[2M + Na]^+^	175.1119[M + H-H_2_O]^+^, 147.1155, 105.0718
**21**	42.1	Butylphthalide (190)	C_12_H_14_O_2_	191.1061[M + H]^+^	191.1067[M + H]^+^	−3.14	213.0872[M + Na]^+^, 403.1888[2M + Na]^+^	173.0989[M + H-H_2_O]^+^, 145.1049[M + H-H_2_O-28]^+^
**22**	42.9	Senkyunolide C (204)	C_12_H_12_O_3_	205.0847[M + H]^+^	205.0859[M + H]^+^	−5.85	227.0685[M + Na]^+^, 431.1431[2M + Na]^+^	187.0763[M + H-H_2_O]^+^, 169.0757[M + H-2H_2_O]^+^, 141.0699[169-28]^+^
**23**	43.4	Senkyunolide E (204)	C_12_H_12_O_3_	205.0843[M + H]^+^	205.0859[M + H]^+^	−7.80	227.0663[M + Na]^+^	
**24**	44.1	Cnidilide (194)	C_12_H_18_O_2_	195.1372[M + H]^+^	195.1380[M + H]^+^	−4.10	217.1185[M + Na]^+^	177.1323[M + H-H_2_O]^+^, 149.1340[M + H-H_2_O-28]^+^
**25**	44.1	*E*-Ligustilide (190)	C_12_H_14_O_2_	191.1061[M + H]^+^	191.1067[M + H]^+^	−3.14	213.0871[M + Na]^+^	
**26**	45.2	*Z*-Ligustilide (190)	C_12_H_14_O_2_	191.1056[M + H]^+^	191.1067[M + H]^+^	−5.76	213.0859[M + Na]^+^, 403.1901[2M + Na]^+^	173.0984[M + H-H_2_O]^+^, 145.0999[M + H-H_2_O-28]^+^, 117.0690
**27**	45.5	Neocnidilide (194)	C_12_H_18_O_2_	195.1372[M + H]^+^	195.1380[M + H]^+^	−4.10	217.1179[M + Na]^+^	177.1323[M + H-H_2_O]^+^, 149.1306[M + H-H_2_O-28]^+^, 121.0998
**28**	45.8	3-Butylidenephthalide (188)	C_12_H_12_O_2_	189.0907[M + H]^+^	189.0910[M + H]^+^	−1.59	211.0720[M + Na]^+^	171.0817[M + H-H_2_O]^+^, 153.0800[M + H-H_2_O-H_2_O]^+^, 143.0882[M + H-H_2_O-28]^+^, 129.0724
**29**	46.2	Riligustilide (380)	C_24_H_28_O_4_	381.2058[M + H]^+^	381.2060[M + H]^+^	−0.52	403.1872[M + Na]^+^, 191.1060, 213.0875	
**30**	48.9	Senkyunolide P (382)		383.2220[M + H]^+^	383.2217[M + H]^+^		405.2031[M + Na]^+^	193.1212[M/2 + H]^+^
**31**	49.2	3′,6,8′,3a-Biligustilide (380)	C_24_H_28_O_4_	381.2058[M + H]^+^	381.2060[M + H]^+^	−0.52	403.1885[M + Na]^+^, 191.1091	
**32**	49.8	Tokinolide B (380)	C_24_H_28_O_4_	381.2037[M + H]^+^	381.2060[M + H]^+^	−6.03	403.1878[M + Na]^+^, 191.1062	
**33**	49.9	Unidentified (382)		383.2220[M + H]^+^	383.2217[M + H]^+^		405.2040[M + Na]^+^	193.1214[M/2 + H]^+^
**34**	50.3	Levistolide A (380)	C_24_H_28_O_4_	381.2064[M + H]^+^	381.2060[M + H]^+^	1.05	403.1887[M + Na]^+^, 191.1054	
**35**	50.8	Senkyunolide O (380)	C_24_H_28_O_4_	381.2061[M + H]^+^	381.2060[M + H]^+^	0.26	403.1879[M + Na]^+^, 191.1057	191.1051[M/2 + H]^+^
**36**	51.4	Senkyunolide M (278)		279.1582[M + H]^+^			301.1402[M + Na]^+^, 579.2952[2M + Na]^+^	
**37**	52.0	Senkyunolide Q (278)		279.1596[M + H]^+^			301.1381[M + Na]^+^, 579.2952[2M + Na]^+^	
**38**	52.7	Unidentified (382)		383.2217[M + H]^+^	383.2217[M + H]^+^	0.00	405.2008[M + Na]^+^	365.2115, 347.1905, 193.1201[M/2 + H]^+^
