# Supplementary material for: Chemical Profiling of an Antimigraine Herbal Preparation, Tianshu Capsule, Based on the Combination of HPLC, LC-DAD-MSn, and LC-DAD-ESI-IT-TOF/MS Analyses
Source: Evid Based Complement Alternat Med. 2014 Jul 20;2014:580745. doi: 10.1155/2014/580745 (PMC4129172; doi:10.1155/2014/580745)
Supplement: Supplementary file 1 — Figure S1 The pharmaceutical manufacture process of TSC described in current Chinese Pharmacopoeia Figure S2. HPLC of 5 batches of TSC samples at 276 nm Figure S3. HPLC of reference solution (RS) and TSC sample. A. RS at 276 nm, B. RS at 221 nm, C. TSC sample at 276 nm, D. TSC sample at 221 nm. Figure S4. HPLC of ethanolic (e) or aqueous (a) extracts of Da Chuanxiong Fang (D.), chuanxiong rhizoma (C.) and gastrodiae rhizoma (G.) at 276 nm. Figure S5. HPLC and TIC of ethanolic extract of Da Chuanxiong Fang. A. HPLC (276 nm), B. (+) TIC, C. (-) TIC. Figure S6. HPLC and TIC of aqueous extract of Da Chuanxiong Fang. A. HPLC (276 nm), B. (+) TIC, C. (-) TIC. [file 580745.f1.doc]

**Supplementary data**

Juanjuan Liang ^1,2^, Huimin Gao ^1,2^*, Liangmian Chen ^1,2^, Wei Xiao ^3^, Zhenzhong Wang ^3^, Yongyan Wang^1^, Zhimin Wang^1,2^*

*1 Institute of Chinese Materia Medica, China Academy of Chinese Medical Sciences, Beijing 100700, China*

*2* *National Engineering Laboratory for Quality Control Technology of Chinese Herbal Medicine, Beijing, 100700, China*

*3 Jiangsu Kanion Pharmaceut Co Ltd, Lianyungang 222001, China*

* Corresponding author. Tel: +86(10)84014128. Fax: +86(10)84014128.

E-mail: [huimin_gao@126.com](mailto:huimin_gao@126.com), zhmw123@263.net


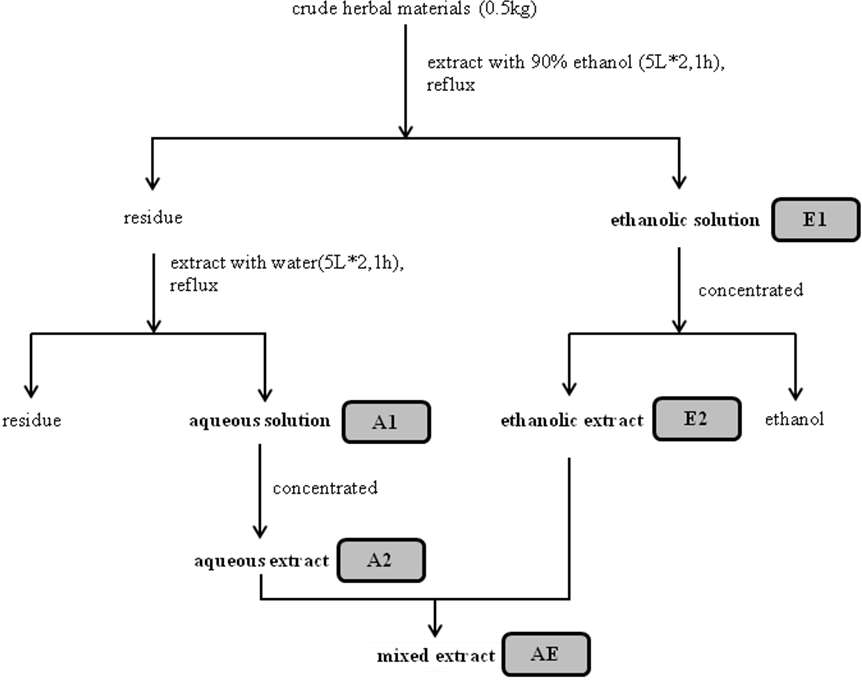


**Figure S1** The pharmaceutical manufacture process of TSC described in current Chinese Pharmacopoeia

**Figure S2**. HPLC of 5 batches of TSC samples at 276 nm

**A**

**B**

**C**

**D**

**Figure S3**. HPLC of reference solution (RS) and TSC sample. A. RS at 276 nm, B. RS at 221 nm, C. TSC sample at 276 nm, D. TSC sample at 221 nm.

**De**

**Ce**

**Ge**

**Da**

**Ca**

**Ga**

**Figure S4**. HPLC of ethanolic (e) or aqueous (a) extracts of *Da Chuanxiong Fang* (D.), *chuanxiong rhizoma* (C.) and g*astrodiae rhizoma* (G.) at 276 nm.

**B**

**C**

**A**

**Figure S5**. HPLC and TIC of ethanolic extract of *Da Chuanxiong Fang*. A. HPLC (276 nm), B. (+) TIC, C. (-) TIC.

**C**

**B**

**A**

**Figure S6**. HPLC and TIC of aqueous extract of *Da Chuanxiong Fang*. A. HPLC (276 nm), B. (+) TIC, C. (-) TIC.

**C**

**B**

**A**

**Figure S7**. HPLC and TIC of ethanolic extract of *chuanxiong* *rhizoma*. A. HPLC (276 nm), B. (+) TIC, C. (-) TIC.

**C**

**B**

**A**

**Figure S8**. HPLC and TIC of aqueous extract of *chuanxiong rhizoma*. A. HPLC (276 nm), B. (+) TIC, C. (-) TIC.

**C**

**A**

**B**

**Figure S9**. HPLC and TIC of ethanolic extract of g*astrodiae rhizoma*. A. HPLC (276 nm), B. (+) TIC, C. (-) TIC.

**B**

**A**

**C**

**Figure S10**. HPLC and TIC of aqueous extract of g*astrodiae rhizoma*. A. HPLC (276 nm), B. (+) TIC, C. (-) TIC.

**B**

**A**

**C**

**Figure S11**. HPLC and TIC of TSC sample obtained using an Agilent 6320 ion-trap spectrometer connected to an Agilent 1200 HPLC system. A. HPLC (276 nm), B. (+) TIC, C. (-) TIC.

**A**

**B**

**C**

**Figure S12**. HPLC and TIC of TSC sample obtained using a Shimadzu LCMS-IT-TOF instrument equipped with a Shimadzu UFLCXR HPLC system. A. HPLC (276 nm), B. (+) TIC, C. (-) TIC.
